# Influence of 3D Printing Parameters on Apparent Resistivity, Repeatability and Time-Dependent Drift of Conductive PLA

**DOI:** 10.3390/polym18111274

**Published:** 2026-05-22

**Authors:** Diana Popescu, Ștefan Cula, Lidia Florentina Parpală

**Affiliations:** Department of Robots and Production Systems, National University of Science and Technology POLITEHNICA Bucharest, 060042 Bucharest, Romania; stefan.cula@upb.ro (Ș.C.); lidia.parpala@upb.ro (L.F.P.)

**Keywords:** carbon black-filled PLA, conductive PLA, material extrusion, printing temperature, layer height, apparent resistivity, repeatability, electrical drift

## Abstract

Conductive filaments for Material Extrusion Additive Manufacturing (MEX) can enable low-cost fabrication of functional parts with embedded electrical features. However, systematic studies on process-dependent electrical properties like apparent resistivity and repeatability are limited, and the post-printing stability of the electrical response is not commonly addressed. This study evaluates the influence of printing temperature, printing speed and layer height on the apparent resistivity, specimen-to-specimen repeatability and time-dependent drift of a commercial carbon black-filled conductive PLA filament (ProtoPasta). The novelty of the study consists of evaluating not only the initial apparent resistivity, but also the repeatability between specimens and the post-print drift of apparent resistivity over a 0–50 h interval. The filament was investigated using three printing temperatures (210–230 °C), two printing speeds (60–80 mm/s) and three layer heights (0.2–0.4 mm), with three replicates per configuration. Apparent resistivity ranged between 0.156 and 0.205 kΩ·mm at t0 and between 0.162 and 0.222 kΩ·mm at t50. Multifactorial ANOVA and main-effects analyses showed that the printing temperature was the main factor affecting mean apparent resistivity at both t0 and t50. Higher temperature reduced apparent resistivity, most likely due to improved polymer flow, inter-bead/inter-layer bonding and conductive-network continuity. Printing speed had no significant main effect on the mean apparent resistivity or drift within the tested range. Repeatability depended on the parameter configuration and measurement time, with variability increasing after 24 h and then becoming mainly dependent on layer height. Drift analysis showed a significant main effect of layer height and a significant layer height × temperature interaction, with the largest increase at 0.3 mm. These results show that parameter selection for conductive MEX parts should consider both the initial resistivity level and post-print stability over time.

## 1. Introduction

The Additive Manufacturing (AM) field has undergone significant advancements, moving beyond its initial applications in prototyping toward the production of end-use parts [1,2], multifunctional devices [1,3] and increasingly complex 3D-printed systems and configurations [4]. AM includes several types of processes, such as vat photopolymerization, powder bed fusion, binder jetting, material jetting, directed energy deposition, sheet lamination and Material Extrusion [5], which differ in feedstock form, material deposition strategy and consolidation mechanism. Among these, Material Extrusion (MEX), also known as Fused Filament Fabrication (FFF) or Fused Deposition Modeling (FDM), and colloquially referred to as 3D Printing (3DP), is nowadays widely used due to its hardware simplicity and affordability, and wide availability of feedstock materials [5,6,7].

Common materials used in MEX include PLA (polylactic acid), ABS (acrylonitrile butadiene styrene), PET/PETG (polyethylene terephthalate/polyethylene terephthalate glycol-modified) and PP (polypropylene), which are selected according to application requirements such as printability, mechanical performance, thermal resistance and chemical resistance [8,9]. At the same time, recent developments have extended this material range toward reinforced, sustainable and functional composite filaments [6]. For instance, natural fiber-reinforced thermoplastic composites have recently been reviewed in terms of their mechanical, thermal and physical properties, showing the diversification of printable thermoplastic composites for MEX [9]. In parallel, conductive composite filaments filled with carbon black (CB), carbon nanotubes (CNT), graphene, metal particles or conductive fibers have enabled the fabrication of electrically functional 3D-printed features, complementing conventional thermoplastic filaments with electrical, thermal or mechanical functionality [7].

Conductive filaments, in particular, are designed to directly integrate electrical functionalities into 3D-printed objects by embedding conductive paths in applications for sensors, antennas, electronic circuits for soft grippers, and microsatellite components [3,10,11]. Thus, the fabrication of electrically functional 3D-printed structures that combine mechanical form and electrical function in a single manufacturing step is enabled. This approach has also the potential of transforming the production of wearables, health monitoring or medical devices [12,13,14]. AM was also investigated for producing conductive sensing elements embedded in composite laminates for structural components. Sam-Daliri et al. [15] embedded carbon nanotube–Elium nanocomposite sensors in unidirectional glass fiber-reinforced epoxy composites for structural health monitoring. In another study, 3D-printed multi-walled carbon nanotube (MWCNT)/epoxy nanocomposite strain sensors were embedded in fiberglass-reinforced laminates for loading monitoring and damage diagnostics [16]. These examples confirm the relevance of 3D-printed conductive materials for sensorized structures, but also show that reliable process–property data on the electrical behavior of printed conductive paths are still needed. Thus, the scientific literature needs to be supplemented with systematic studies in order to guide the efficient development of such products, which is an objective of this research.

Current studies showed that the electrical conductivity of filaments incorporating carbon-based fillers such as CB, graphene, CNT or milled CFs is much lower than that of traditional metallic conductors like copper or silver [17]. Moreover, the electrical performance of 3D-printed features is not determined only by the filament composition. The values of process parameters such as printing temperature, printing speed, layer height, nozzle diameter or print orientation directly affect the internal microstructure of 3D-printed parts [18,19,20,21]. These parameters influence the deposited-path geometry, inter-bead and inter-layer contact quality, and void formation, which in turn affects the resistivity and reliability of conductive features [18,20,22].

Additionally, considering the specificity of the MEX process where objects are built from adjacent beads of material and by superposing layers, the electrical response is not determined only by the intrinsic resistivity of the filament, but also by the quality of the interfaces, i.e., the region of contact between beads and layers, which are related to the cross-section geometry given by layer height and bead width. Each such interface can act as a barrier to the electrical charge transport, impacting the effective conductivity of the printed path. So, the overall resistivity of the 3D-printed path depends on both filament composition and the degree of inter-layer bonding obtained by controlling different process parameters. In this research, the electrical performance is reported as apparent resistivity, based on two-probe resistance measurements using the nominal gauge length and cross-sectional area of the 3D-printed paths. This metric aims to capture the combined effects of intrinsic material resistivity and inter-bead/inter-layer contact quality, thus being directly relevant for functional 3D-printed conductive features.

While higher printing temperatures are reported to generally improve the conductivity by improving polymer flow and inter-layer bonding [21], as mentioned, other process parameters such as layer height, printing orientation, bead orientation and infill density can also play a role. Pentek et al. investigated process parameters’ influence on the electrical and mechanical properties of PLA- and ABS-based carbon composites [19]. Beniak et al. examined the effects of nozzle temperature, printing speed, layer height, infill pattern and density on the electrical resistance and tensile strength of ProtoPasta conductive PLA [23]. Stano et al. [18], focusing on the same commercial filament, reached the conclusion that printing temperature and layer height significantly influence the resistivity, a finding also reported in [23]. Gao et al. [24] studied ProtoPasta and BlackMagic filaments, and showed that for ProtoPasta, the nozzle temperature and printing speed significantly affect resistivity, with temperature showing a parabolic relationship, as confirmed by statistical analysis trends. For the graphene-based filament, printing temperature was the only statistically significant factor affecting the resistivity [24]. Glogowsky et al. [25] explored the effects of MEX parameters like print speed, temperature, infill orientation, and layer height on the conductivity of elastomer filaments with carbon-based fillers. Naboulsi et al. [26] investigated the impact of printing temperature on the conductivity and structure of PLA-40%CB, and identified an optimal range (210–230 °C) that improved the conductivity for the studied specimen. There are also studies presenting in-house conductive filaments [27,28,29], which are developed to address the low conductivity of the commercially available options.

As a consequence of these studies, several general guidelines are provided: 3D printing at higher, filament-compatible temperatures usually improves conductivity, while adjusting the printing speed and layer height allows for fine-tuning electrical performance. However, there is a lack of systematic data on the particular parameter settings and electrical properties of different conductive filaments, particularly PLA-based, which are of interest in this research for their affordability and ease of use, with a potential of increased use for producing functional products. Moreover, data on post-print time dependence and specimen-to-specimen repeatability for the same parameter setting remain scarce, which limits the reliability of process-related property guidelines for functional parts.

In this context, the purpose of the current research was to answer the following questions. RQ1: How do MEX parameters (printing temperature, speed and layer height) influence the apparent resistivity level of PLA-based conductive ProtoPasta? RQ2: How consistent are the 3D-printed replicates for the same parameter setting (specimen-to-specimen repeatability)? RQ3: How does apparent resistivity evolve over time after printing (0–50 h), and which process parameters control the drift magnitude?

To address these RQs, a factorial experimental design was conducted using a commercially available CB-filled conductive PLA filament (ProtoPasta), by varying printing temperature, speed and layer height. Three aspects, relevant for functional conductive parts, were considered: apparent resistivity level, specimen-to-specimen repeatability for identical parameter settings, and time-dependent drift of apparent resistivity 0–50 h after printing. By quantifying these effects, the work provides experimental data on the stability of the electrical response of conductive MEX structures and contributes to more reliable process–property guidelines.

## 2. Materials and Methods

**Figure 1:**
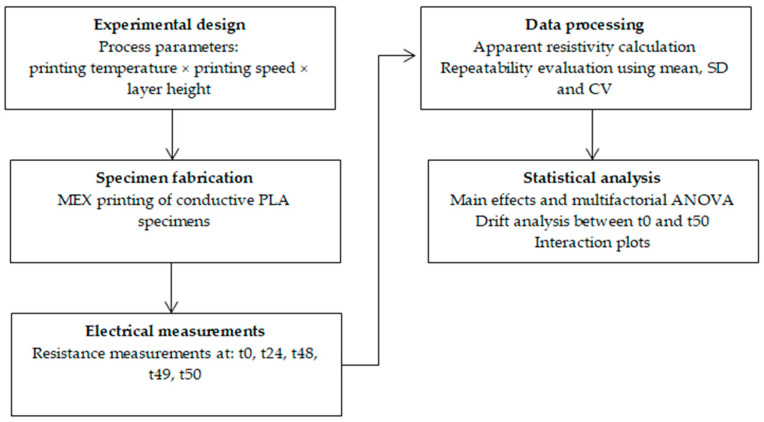
Flowchart of the experimental procedure used in the study.

The following research steps (Figure 1) were used to evaluate how the selected MEX process parameters impact the electrical response of ProtoPasta filament (ProtoPlant Inc., Vancouver, WA, USA):

- 3D-printed specimen fabrication. The specimens were manufactured using three levels of printing temperature, two levels of speed and three levels of layer height, based on a factorial plan. Three replicates were manufactured for each configuration.
- Electrical measurements and measurement variability evaluation. The electrical resistance of all specimens was measured at predefined time points (t0, t24, t48, t49, t50), and apparent resistivity was calculated for further use in analysis.
- Statistical analysis. Multifactorial ANOVA was applied to evaluate the main effects of the process parameters on apparent resistivity and on the time-dependent drift between the initial (t0) and final (t50) states. Interactions between factors were explored graphically using interaction plots.

### 2.1. Fabrication of 3D-Printed Conductive Specimens

Table 1 summarizes the electrical properties reported by the manufacturer [30], alongside values extracted from the literature for the same conductive PLA filament. The investigated material was ProtoPasta conductive PLA based on a NatureWorks 4043D PLA matrix filled with CB (reported at approximately 20–25 wt%).

Manufacturer’s recommended process parameters were used to set the 3D printing profiles in OrcaSlicer v.2.3.1, open-source slicing software developed by the OrcaSlicer open-source community. The generated G-code was used on the Comgrow T300 3D printer (Sovol, Shenzhen, China). All conductive specimens were 3D-printed with the filament paths oriented parallel to the electrical contacts, as previous studies have shown that this infill orientation improves the electrical conductivity [31].

**Table 1:** ProtoPasta electrical properties and recommended 3D printing process parameters.

Electrical Properties ProtoPasta—Manufacturer [30]	Recommended Process Parameters [30]	Data from the Literature
Volume resistivity: 15 Ω·cm Along the layers (*x*/*y*-axis): ~30 Ω·cm Across the layers (*z*-axis): ~115 Ω·cm Electrical resistance 2.5 to 3.5 kΩ for 100 mm length	Printing temperature: 210–230 °C Bed temperature: 60 °C Printing speed up to 100 mm/s Extrusion multiplier: 100% Fan: 100%	Raw filament resistivity: 3.1–5.1 Ω·cm [32] Printed monolayer structures at 0°: 8.2–15 Ω·cm, depending on layer thickness [32] Reported electrical resistance values for printed specimens: 0.89–6.85 Ω, depending on length, nozzle temperature and measurement temperature [23] Printed specimen resistivity: 15.36–16.38 Ω·cm (four-probe); 16.79–62.37 Ω·cm (two-probe) [33]

One can note that the values in Table 1 show considerable dispersion. This variability can be attributed to differences in specimen geometry and measurement method (two-probe vs. four-probe), as well as print orientation and infill strategy.

Printing temperature, printing speed and layer height were selected as parameters of interest considering their influence on the electrical conductivity of conductive PLA filaments, as suggested by the literature data, material properties and printing process. Printing temperature has a significant impact on resistivity, as discussed in several other studies [10,23,24,25,26], with higher temperatures improving polymer flow and inter-layer bonding, and thus lowering the resistivity. Printing speed also affects electrical properties by influencing the quality of layer adhesion and the consistency of material deposition [23,24,25]. Layer height impacts the contact between the layers, the void formation, as well as the distribution of conductive fillers [10,18,25].

The parameter levels for this study are summarized in Table 2.

The temperature range (210–230 °C) and printing speeds (60–80 mm/s) were selected within the limits recommended by the manufacturer. Layer height values of 0.2 mm, 0.3 mm and 0.4 mm were chosen as they represent practical levels in MEX, allowing the evaluation of layer height influence on electrical performance.

The conductive paths were intentionally printed as simple rectangular specimens, rather than as complete sensor structures, because the objective was to isolate the effect of printing temperature, printing speed and layer height on the electrical response of the printed path itself.

### 2.2. Electrical Measurements

#### 2.2.1. Experimental Setup

The initial measurements (t0) were performed after the specimens had cooled to room temperature following 3D printing and removal from the build plate. Subsequent measurements were performed after 24 h (t24), 48 h (t48), 49 h (t49) and 50 h (t50).

**Figure 2:**
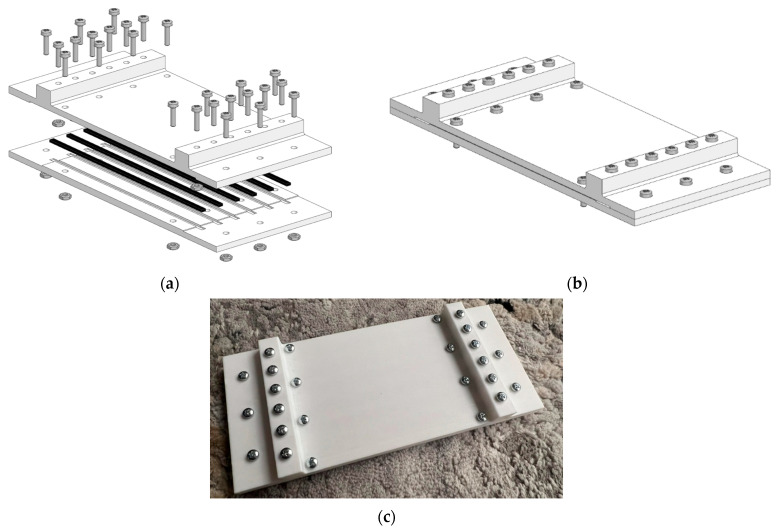
Deconstructed view of assembly (**a**), mounted assembly (**b**), physical assembly (**c**).

**Figure 3:**
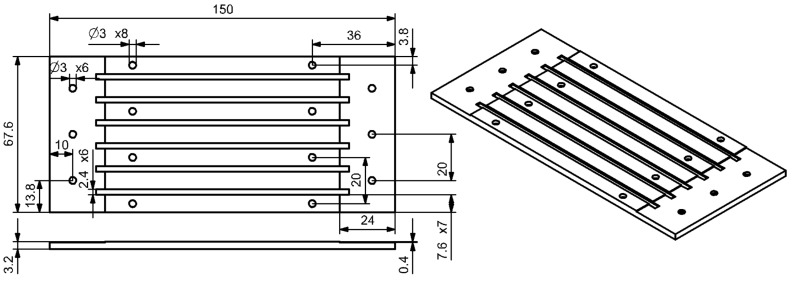
Base view and dimensions; all dimensions are in mm.

**Figure 4:**
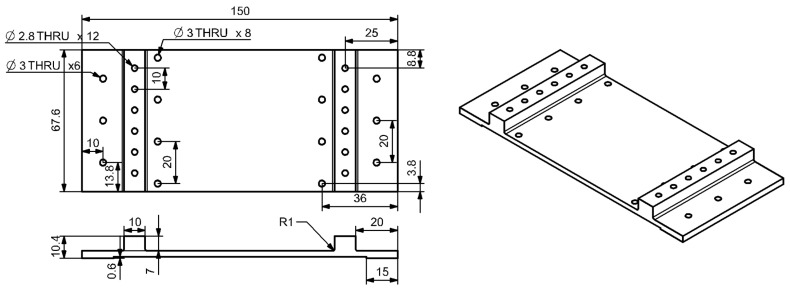
Lid view and dimensions; all dimensions are in mm.

To minimize the measurement variability caused by probe repositioning, inconsistent contact pressure or mechanical strain effects, a custom 3D-printed holder was designed and manufactured from standard PLA using the same 3D printer as for the specimens. The holder (Figure 2) provided a constant distance between measurement electrodes, as well as repeatable compression of the conductive paths during measurements. It consisted of a base (Figure 3) and a lid (Figure 4). The base included channels for positioning six conductive paths, clearance holes for electrode screws, M2.5 × 10 mm and embedded hex nuts for assembly. The resistance contribution of the metal screws was assumed negligible in comparison to the 3D-printed conductive paths.

The lid provided alignment and ensured repeatable clamping of the conductive paths during measurement. After 3D printing, the conductive paths (see Section 2.2.2) were placed in dedicated channels in the base (Figure 3). These channels ensured lateral positioning and prevented specimen displacement during clamping.

The spacing between individual paths (7.6 mm) was calculated considering the M2.5 screw head dimensions (approximately 4 mm) plus the space for the multimeter alligator clip prongs, thus ensuring the required isolation between the conductive paths. We used 3 mm diameter clearance holes, the 0.5 mm diametral clearance accommodating the typical MEX tolerances (±0.2 mm) while providing operational room for easy insertion and thread engagement with embedded nuts.

The lid (Figure 4) was the counterpart of the base. On the bottom side of the lid, on its left and right extremities, two 0.6 mm spacers were added to provide contact with the holder base when assembled with the conductive path between them. This created a fixed reference plane that prevented the lid from bending. It also ensured that the pressure applied after assembly is uniform on the paths, thus further isolating external factors which could influence resistivity.

No specific ASTM or ISO standard was used for the electrical measurement protocol, as the study focused on comparative apparent resistivity and post-print drift of custom MEX conductive paths. The same specimen geometry, holder design, electrode configuration, and measurement procedure were used for all parameter combinations to ensure comparability.

**Figure 5:**
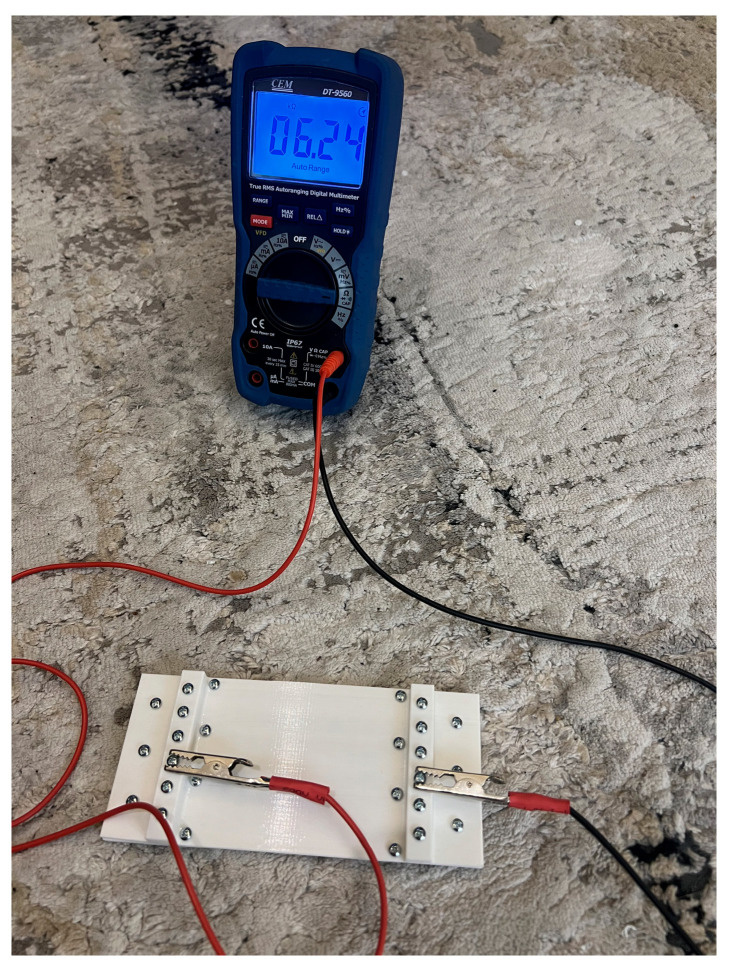
Physical assembly during measurement process.

Electrical resistance was measured using a two-probe configuration with a CEM DT-9560 digital multimeter (accuracy ±0.8% + 1 digit). The multimeter probes were connected to the electrode screws (Figure 5), which established contact with the conductive paths. The instrument applies an internal test current of about 1–2 mA, and calculates resistance according to Ohm’s law. A two-probe configuration was used because the objective was to evaluate the apparent resistivity of functional 3D-printed conductive paths under repeatable connection conditions, rather than the intrinsic bulk resistivity of the filament material. The two-probe method also corresponds to how the printed conductive paths are connected in simple functional circuits, where the measured electrical response includes the printed path, inter-bead/inter-layer interfaces and electrode contact regions. Moreover, the same holder design, electrode spacing, clamping method and measurement procedure were used for all specimens, which allowed comparative evaluation between process parameter combinations.

Nine identical holders were used in the experiments. Each holder contained six conductive paths, corresponding to three replicates for each of the two printing speeds. Within each holder, temperature and layer height were fixed, while printing speed varied between specimens.

Apparent resistivity, *ρ*, was calculated using Equation (1):

(1) $$\rho =R*\frac{A}{L}$$

where *R* is the measured resistance, *A* is the cross-sectional area of the conductive path and *L* is the nominal gauge length between measurement electrodes.

#### 2.2.2. Electrically Conductive Specimens

**Figure 6:**
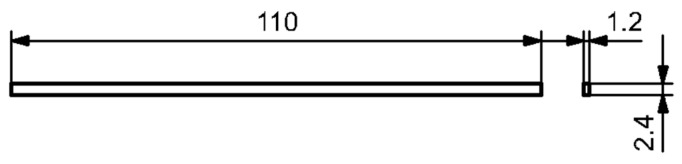
Dimensions of electrically conductive specimens; all dimensions are in mm.

The electrically conductive specimens were designed as rectangular bars measuring 110 mm × 2.4 mm × 1.2 mm (Figure 6). The nominal gauge length between the electrode centerlines was 100 mm. The extra length compensated for the 3D printer acceleration and deceleration at the path ends, so that the middle of the path was 3D-printed at constant speed. This also helped limit the measurement variability due to filament corner bulging, mainly caused by excessive nozzle pressure when changing direction in sharp corners.

The specimens were placed in OrcaSlicer as six individual objects per build plate. For each batch, the printing temperature and layer height were kept constant, while the printing speed was assigned differently to the specimens in order to obtain three specimens at 60 mm/s and three specimens at 80 mm/s. The conductive paths were oriented parallel to the measurement direction, so that the deposited filament lines were aligned with the electrical current path [31]. The G-code was then checked using the layer simulation before printing. The 2.4 mm width allowed the printer to do exactly six passes with a 0.4 mm nozzle, ensuring 100% line infill. This geometry also provided a stable contact area for the M2.5 electrode screws used during measurements, while the 1.2 mm height was selected as the lowest common multiple of the investigated layer heights (0.2, 0.3 and 0.4 mm).

**Figure 7:**
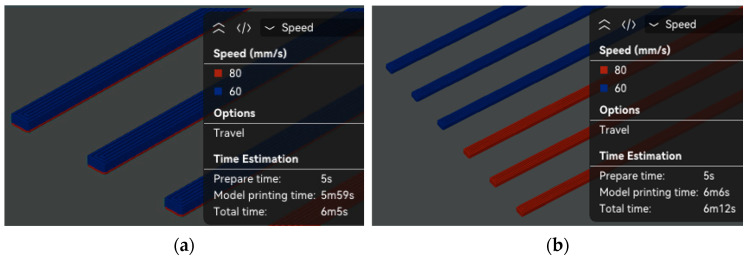
Before G-code modification (**a**) and after manually modifying the first layer speed (**b**).

**Figure 8:**
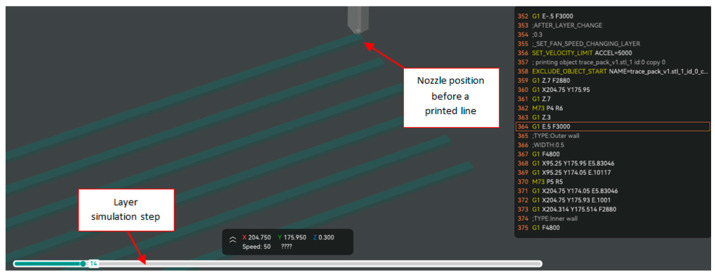
Layer simulation before the first printed line of a specimen.

**Figure 9:**
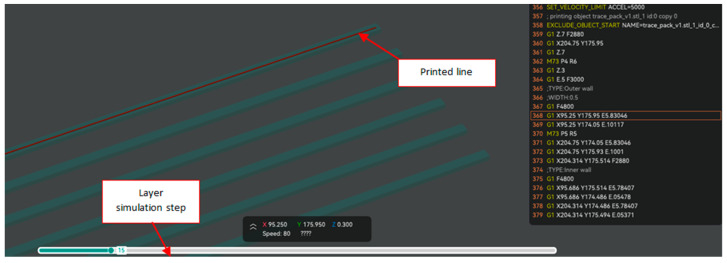
Layer simulation after the first printed line of a specimen.

Due to OrcaSlicer limitations related to per-object settings, different first-layer heights and temperature changes during the same print could not be assigned. Additional manual G-code modifications were also necessary because the first-layer speed could not be modified through per-object settings (Figure 7). Therefore, the commands corresponding to the first-layer feed rate of the 60 mm/s specimens were changed from G1 F4800 (80 mm/s) to G1 F3600 (60 mm/s), as illustrated in Figure 8 and Figure 9. In practice, the G1 F4800 commands were located around the first 3D-printed line of each specimen, and the feed rate value was changed accordingly.

The modified options are related to the speed settings in OrcaSlicer (Table 3), which allow modification of either global settings or per-object settings. The first three specimens used per-object speeds, while the remaining three were printed using the modified global settings.

## 3. Results and Discussion

The results were processed according to the RQs, and are reported as follows. As the apparent resistivity, calculated using Equation (1), changed over time, the measurements performed at t0, t24, t48, t49, and t50 were used to evaluate both repeatability and temporal drift. Section 3.1 investigates the specimen-to-specimen repeatability at each considered time point. Section 3.2 investigates the main effects and interactions of the process parameters on apparent resistivity using Minitab Statistical Software 22.5.1. The analysis was performed at two time points, t0 and t50, in order to compare as-printed behavior with a final measured state. Section 3.3 presents the time-dependent drift which quantifies the evolution of the apparent resistivity with time, and evaluates the magnitude of the change for each configuration.

Table 4 presents the results of apparent resistivity at t0. The data for the other time intervals are reported in the Supplementary Material. In Table 4, 60-1 denotes the coding of the first specimen (out of three replicates per configuration), 3D-printed at 60 mm/s.

### 3.1. Repeatability Across Specimens over Time (t0–t50)

Repeatability was assessed based on the measurements of the three replicates 3D-printed for each configuration. All results are provided in the Supplementary Material, while Table 5 summarizes the measurements taken at t0, separated by print speed values.

**Figure 10:**
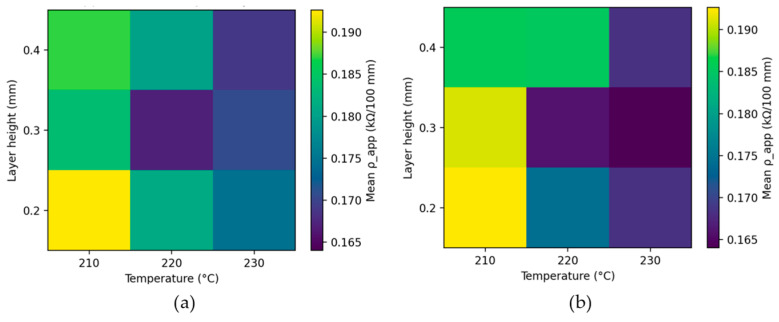
Mean apparent resistivity for 60 mm/s (**a**) and 80 mm/s (**b**) specimens at t0.

To visualize the apparent resistivity distribution, mean values are presented as heat maps on a temperature–layer height grid, with printing speed shown in separate panels for 60 mm/s and 80 mm/s (Figure 10).

**Figure 11:**
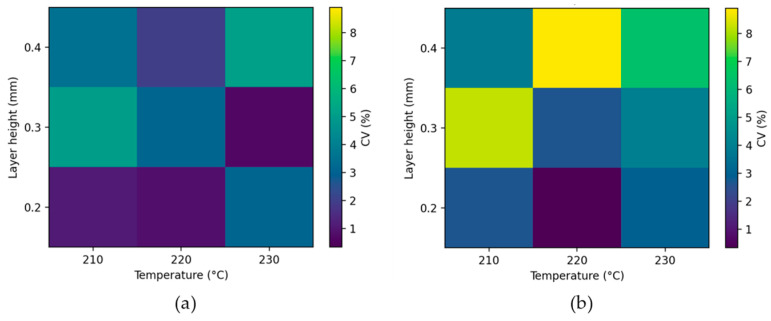
Coefficient of variation (CV) of apparent resistivity at t0 for specimens printed at 60 mm/s (**a**) and 80 mm/s (**b**).

Specimen-to-specimen repeatability was evaluated using the coefficient of variation (CV, %) of apparent resistivity, plotted using the same temperature–layer height grid and separated by printing speed (Figure 11).

At t0, the specimens manufactured at 80 mm/s printing speed had a low dispersion range (CV ≈ 2.6–4.0%) for the most configurations (see also Supplementary Material). However, three specimens clearly showed a higher variability, i.e., configurations: 210 °C/0.3 mm (CV = 8.23%), 220 °C/0.4 mm (CV = 8.90%), 230 °C/0.4 mm (CV = 6.41%). The best repeatability was obtained at 220 °C/0.2 mm (CV = 0.33%). When analyzing the configurations with a high CV, it could be noticed that the spread was caused by one replicate which deviated from the others. This suggested a potential process-sensitive zone at higher layer heights. At 60 mm/s printing speed, the repeatability was more uniform between configurations. The 230 °C/0.3 mm and 220 °C/0.2 mm configurations had almost an identical CV (0.68%, respectively 0.84%), while the other configurations had a CV ≈ 1.08–5.12%. The highest variability was recorded for 230 °C/0.4 mm (CV = 5.12%) and 210 °C/0.3 mm (CV = 5.06%). In general, the specimens measured at t0 showed more robust reference repeatability at 60 mm/s.

It also should be noted that the outlying specimen was not always the same over time, between replicates. In some cases, one specimen constantly deviated, while in others the outlier changed with time. This suggested a time-dependent drift rather than a defect in one single replicate.

After 24 h, the specimen-to-specimen repeatability became increasingly dependent on the layer height parameter. When compared with the measurements at t0, the unstable configurations changed at t24, and no advantage of 60 mm/s over 80 mm/s could be noted anymore. The highest CV values were recorded for specimens with 0.3–0.4 mm layer height, up to 11.11%. After 48–50 h, instability was observed for 0.3 mm layer height specimens, especially at 60 mm/s, where the CV reached the largest values (14.96%), while 0.2 mm layer height remained the most stable across temperatures (CV ≤ 2%). At 80 mm/s, the instability remained localized, mainly at 0.3 mm layer height for 210–220 °C, where CV values were around 11.6%, whereas the 230 °C/0.3 mm configuration showed much lower dispersion. These results indicate that time enhanced the parameter-sensitive variability, with 0.3 mm layer height being the most frequent source of poor repeatability after 24 h.

Thus, the variability analysis suggested that the specimens’ repeatability, in terms of electrical performance, depends on the interaction between process parameters and time. At t0, the printing speed made the difference between the results. However, at 24 h, the speed effect was less relevant; instead, variability became related to the layer height parameter. These descriptive repeatability results suggested that the variability is more parameter-sensitive over time, with 0.3 mm layer height being the most frequent source of poor repeatability after 24 h. From 48 h onward, layer height seems to be the main descriptive factor associated with a higher dispersion. The configurations with 0.2 mm layer height were the most stable, especially at 60 mm/s, while a layer of 0.3 mm produced unstable specimens at both analyzed speeds, with the largest dispersion observed at higher printing temperatures.

Among the investigated configurations, 220 °C/60 mm/s/0.4 mm provided the most favorable combination of low apparent resistivity and stability over the 0–50 h interval.

One can interpret the observed behavior by considering the percolative nature of electrical conduction in the ProtoPasta filament, where conductivity is enabled by the CB-based fillers, as suggested by the literature [17,21,34]. The electrical transport depends on the formation of continuous conductive pathways during filament deposition and solidification, as well as on how robust the network formed in the 3D-printed structure is. Thus, the apparent resistivity is controlled by filler content, particle distribution established during the extrusion process, the number of particle–particle contacts, and the presence of gaps between particles. All these can enable or limit tunneling transport. In MEX, the electrical response is also dependent on interface continuity established between beads and layers. Small differences in the fusion quality between beads and layers, local interface defects or voids [21] can determine large changes in the measured resistivity. Moreover, relaxation of the PLA matrix after 3D printing can also modify weak conductive contacts, contributing to the time-dependent drift and configuration-dependent repeatability observed in the measurements.

The process parameters influence the electrical conduction in a different manner. Printing temperature mainly controls the melt viscosity and inter-bead fusion. Thus, at higher temperature, the beads fuse better, increasing the contact area and probability to establish bridges between conductive fillers. This makes the percolation network more redundant and lowers its resistivity. At lower temperature, the fusion is weaker and conduction becomes more interface-limited, with inter-road boundaries acting as weak links. Temperature can also change how CB fillers are distributed during the extrusion flow in the nozzle, as lower viscosity changes the shear-driven dispersion and the spacing between CB particles. As conduction is sensitive to how close the particles are, these effects shift the effective percolation state. At lower and intermediate temperatures, such as 210–220 °C, the network may be only marginally connected, so small variations in fusion or local geometry can cause large specimen-to-specimen differences. The results of the current research are consistent with other studies [18,23,26,34] in terms of temperature’s impact on resistivity, the percolation behavior being reported depending on printing temperature.

The layer height was found to be the most important factor in the time-dependent study. At 0.2 mm, more layers are deposited to build the specimens, thus increasing the number of inter-layer contacts and creating multiple parallel conductive paths, and making the resistance more stable. At 0.3 mm, the 3D-printed specimens were found to be in a transition zone, with fewer interfaces than at 0.2 mm layer height. If this connectivity is close to the percolation threshold, small post-print changes (matrix relaxation or interfacial rearrangement) can open or break the bridges between the conductive fillers, and impact the specimen-to-specimen variability. At higher layer height, porosity may also increase, thus reducing the effective conductive cross-section and introducing bottlenecks.

Printing speed has an impact on how consistent the conductive network is formed during deposition. At higher speed (80 mm/s), there is less time for the melted filament to spread and for inter-bead/inter-layer fusion. This can increase the micro-voids, incomplete bonding, and variability in contact area. Higher printing speed also increases shear in the nozzle that can modify CB filler spacing. At 80 mm/s, even minor deposition defects or instabilities can create local weak regions that have a very large impact on the measured resistance.

The intermediate time points of t24, t48 and t49 were used to describe the evolution of specimen-to-specimen repeatability based on mean, SD and CV values, while statistical testing was focused on the initial state (t0), the final state (t50) and the calculated drift between t0 and t50. Thus, the trends noted at the intermediate time points should be interpreted as descriptive repeatability trends.

### 3.2. Main-Effect Analysis of Process Parameters on Apparent Resistivity

For the main-effects analysis, two time points were considered: t0 (the initial post-print electrical response) and t50 (the final state after the 50 h monitoring interval). As cumulative post-print changes were expected to be largest at t50, a comparison was made for evaluating if the influence of the process parameters on apparent resistivity changed over time.

**Figure 12:**
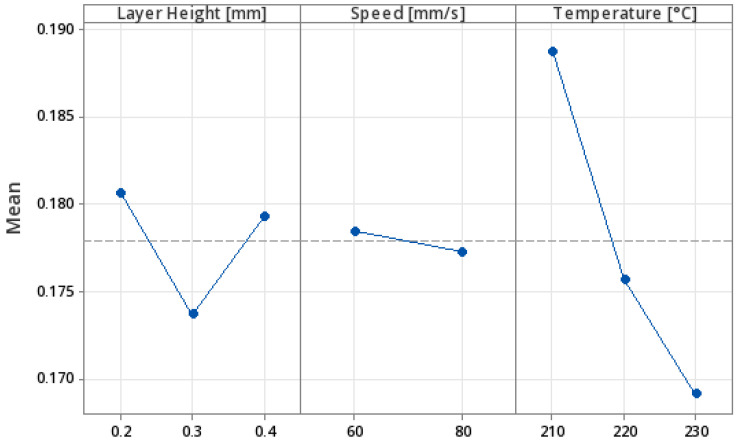
Main-effects plot for apparent resistivity at t0.

Figure 12 shows the main-effects plots based on measurements at t0. It can be noted that the temperature had the dominant effect. The mean apparent resistivity decreased strongly from 210 °C → 220 °C → 230 °C. The vertical separation between the temperature-level means was clearly larger than for the other factors, showing that temperature is the main parameter influencing the resistivity level at t0.

Layer height had a U-shaped trend. The mean was higher at 0.2 mm, reaching its minimum at 0.3 mm, and then increasing at 0.4 mm layer height.

Printing speed had a small effect on the mean values of apparent resistivity, the line being almost horizontal as can be noted in Figure 12, while temperature is the relevant parameter.

At t0, the ranking of the process parameters can be explained by the way the conductive network was formed during deposition. Temperature has the strongest effect because it improves interfacial continuity and particle proximity. Layer height modifies the network geometry and leads to an optimum within the tested range, while printing speed mainly affects robustness rather than the mean resistivity level.

All these observations were in line with ANOVA results (Table 6).

**Figure 13:**
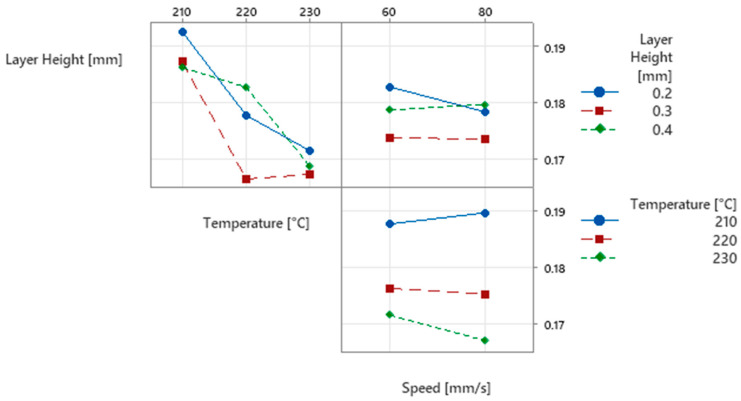
Parameter interaction plots at t0.

The interactions between parameters at t0 are shown in Figure 13. For the combination temperature × layer height, the plots suggest a possible interaction because the lines corresponding to different layer heights are not perfectly parallel. This is in line with the findings of Stano et al. [18]. For all layer heights, the apparent resistivity decreases with increasing temperature. However, the size of this decrease differs between layer heights, indicating that the temperature effect is modulated by the layer height. The other two interactions, speed × layer height and speed × temperature, showed weak interaction, the lines being almost parallel.

The main conclusion at t0 is that the printing temperature is the dominant factor for the resistivity, followed by layer height which presents a non-monotonic optimum around 0.3 mm. Printing speed does not have a main effect on the mean within 60–80 mm/s. A similar temperature effect over electrical resistance was reported for ProtoPasta conductive PLA [18,23], where higher nozzle temperature produced lower resistance levels and was statistically significant in ANOVA.

**Figure 14:**
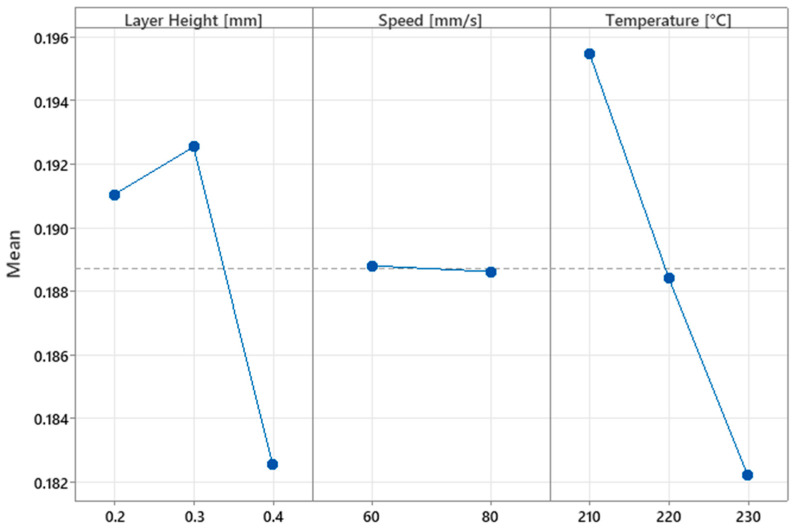
Main-effects plot for apparent resistivity at t50.

At t50, the mean apparent resistivity (Figure 14) still showed a clear dependence on printing temperature, decreasing from 210 °C to 230 °C. Layer height also changed the response pattern, although its main effect was not statistically significant.

Table 7 presents the ANOVA results for t50.

The printing speed effect on the mean was not significant for the investigated values (60 mm/s, 80 mm/s), with small differences between the two levels, which suggests that speed mainly influences variability. However, the layer height ranking changes when comparing t50 and t0. At t50, the mean apparent resistivity is highest at 0.3 mm, intermediate at 0.2 mm, and lowest at 0.4 mm, i.e., in a reverse pattern than for t0. This showed that time-dependent effects are not uniform across layer heights. Thus, the layer height that minimizes resistivity immediately after printing may differ from the one that minimizes resistivity in time. This was one of the reasons for a deeper analysis of the drift in Section 3.3.

**Figure 15:**
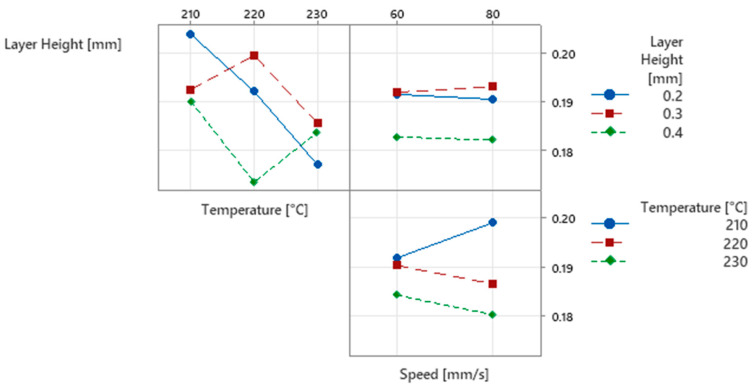
Parameter interaction plots at t50.

The interaction plots at t50 (Figure 15) indicated that the temperature × layer height combination had a more visible interaction than at t0. The 0.4 mm level, especially, showed a different response across temperature compared with 0.2–0.3 mm, suggesting that temperature sensitivity is dependent on layer height after 50 h. In comparison, speed × layer height and speed × temperature presented almost parallel lines, and small separations between 60 and 80 mm/s, suggesting weak interaction.

In general, from t0 to t50, the temperature remains the dominant factor and print speed has little effect on the mean resistivity, whereas layer height changes its main-effect pattern and the temperature × layer height interaction becomes more pronounced. This suggests that, after 50 h, the electrical response depends not only on the initial conductive network formed during 3D printing, but also on how stable this network remains over time.

From a practical perspective, optimizing process parameters for minimum resistivity immediately after printing does not guarantee minimum resistivity after conditioning; therefore, parameter selection for conductive MEX parts should include time-dependent performance criteria (drift) in addition to initial values.

### 3.3. Time-Dependent Apparent Resistivity and Drift Characterization

A new data set was generated from the results including Δ*ρ*% (calculated with Equation (2)) and the response column drift, while keeping the three process parameters as factors.

(2) $$∆\rho =100*\frac{\rho_{t50}-\rho_{t0}}{\rho_{t0}}$$

A factorial ANOVA was conducted (Table 8) on the 54 individual Δ*ρ*% values (three replicates for each of the 18 parameter combinations). The model included the three main effects and all two-factor interactions. The three-way interaction was computed in a preliminary model and it was not statistically significant, thus it is not presented in the final reported model.

As the measurements were performed using metallic screws as fixed contact electrodes, the local effects at the screw–composite interface may have contributed to measurement scatter, especially through mechanical relaxation of the PLA-based matrix around the contact area. For this reason, the drift was analyzed at the specimen level using all 54 individual Δ*ρ*% values, and interpreted mainly through consistent trends across process conditions, rather than through isolated outliers.

ANOVA results showed that drift was significantly influenced by the layer height parameter (*p* = 0.000274) and by the layer height × temperature interaction (*p* = 0.000038). By comparison, the printing speed had no significant main effect on drift, and the interactions involving printing speed were not significant either. The main effect of temperature was not statistically significant at a 0.05 level, although it showed a tendency toward significance (*p* = 0.0738).

The significant layer height × temperature interaction showed that the time-dependent drift cannot be attributed just to the layer height parameter. Instead, the magnitude and direction of Δ*ρ*% depended on the specific combination of layer height and printing temperature. The mean drift was highest at 0.3 mm layer height, but this effect was especially pronounced at 220 °C and 230 °C. In comparison, the 0.4 mm layer height had a smaller average drift, with negative drift values observed for the 220 °C/0.4 mm configuration. Therefore, the drift over 0–50 h depended on both layer height and printing temperature.

These results answered RQ3 by showing that the time-dependent drift in apparent resistivity over time (0–50 h) is controlled mainly by layer height and by the layer height × temperature interaction. This was in contrast with the results at t0, where the printing temperature mainly determined the initial apparent resistivity level. Therefore, the parameters that minimize initial resistivity are not necessarily the same parameters that provide the best post-print electrical stability.

The time-dependent evolution of apparent resistivity can be related to the stability of the conductive path. In CB/PLA materials, the electrical response depends on filler dispersion, but also on the bonding between deposited lines and layers [21,22]. After printing, the polymer matrix may still relax, and residual stresses may decrease, and these small changes can modify some conductive links and produce changes in resistance. Similar resistance variations were also reported for MEX conductive filaments in other studies [18]. This suggests that the conductive network can still change after 3D printing. The increase in variability from 24 h to 50 h was consistent with this behavior. During this period, the relaxation of the PLA matrix and of the interfaces between deposited lines and layers may slightly change particle spacing and conductive contacts, and these changes can affect the stability of the conductive bridges.

The higher mean drift at 0.3 mm suggests that this layer height produced a less stable conductive network. The network probably had fewer alternative conductive paths and depended more on inter-layer contacts. Therefore, small post-print changes could more easily affect the electrical response. This can explain the larger positive Δ*ρ*% values and the higher specimen-to-specimen variability.

By comparison, the lower drift at 0.2 mm layer height was consistent with the better interfacial continuity obtained with thinner layers. At 0.4 mm, the average drift was smaller. The negative values observed in some cases, especially at 220 °C, may indicate partial stabilization or rearrangement of conductive pathways.

Compared to prior studies [18,23,26], the present results confirm that printing temperature is a major factor reducing the apparent resistivity of conductive ProtoPasta prints. However, our findings also showed that the initial temperature effect is not sufficient to define the process window. When separately analyzing the initial apparent resistivity, specimen-to-specimen repeatability and post-print drift over 0–50 h, it was shown that the parameters controlling the initial resistivity level are not necessarily the same as those controlling post-print electrical stability.

## 4. Case Study

### 4.1. Experimental Setup

The case study investigated how the length of 3D-printed conductive paths affects the light intensity of light-emitting diodes (LEDs) powered through those paths. It was intended as a simple application-level illustration of the voltage drop associated with printed conductive path resistance, not as a separate statistical experiment.

**Figure 16:**
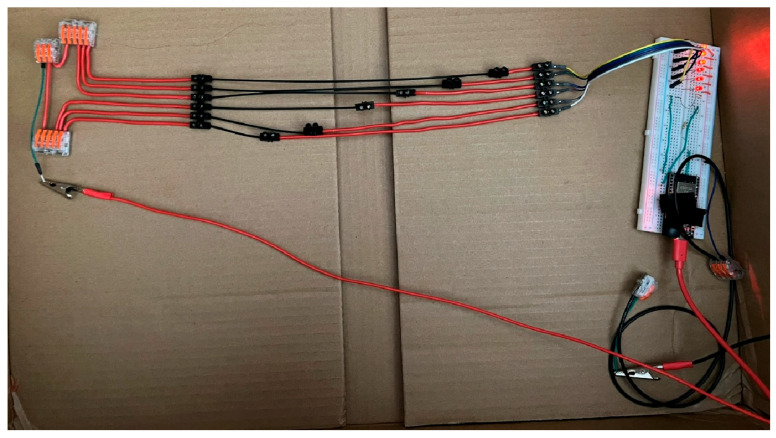
Assembled setup.

The measurement setup (Figure 16) included an ESP32 development board that read the analog output of a photoresistor through its 12-bit ADC, six LEDs each powered through a separate conductive path of different lengths, and a photoresistor positioned in front of the LED connected through the investigated conductive path. A laboratory power supply provided a constant 5 V, while Wago (WAGO GmbH & Co. KG, Minden, Germany) connectors and a terminal strip distributed power and interface conventional wiring with the printed conductive paths.

The conductive paths were 3D-printed from the ProtoPasta spool used in the study, using the parameter combination selected from the main experimental study for its favorable balance between low apparent resistivity and stability, namely 220 °C/60 mm/s/0.4 mm. Their nominal lengths were 60, 100, 140, 180, 220, and 260 mm, providing a gradual progression from the shortest to the longest path. Each path included an additional 10 mm length to compensate for bends and routing features, so that the comparison between paths was not biased by layout artifacts. The different path lengths were used to show that the electrical behavior measured on straight specimens can affect a simple printed circuit. Longer conductive paths have higher resistance and therefore can reduce LED brightness.

The ESP32 development board acted as the central control and acquisition unit, sampling the photoresistor output through its 12-bit ADC (range 0–4095), which was used as a relative indicator of light intensity. The LED array, 3D-printed paths, and photoresistor were then placed inside an opaque enclosure so that ambient light did not influence the readings. The photoresistor was positioned in front of the corresponding LED, and measurements were carried out successively for the investigated conductive path lengths. The ESP32 subsequently recorded the signal every 15 s over a sufficiently long interval to capture both short-term fluctuations and slow changes in light intensity.

### 4.2. Data Processing and Visualization

**Figure 17:**
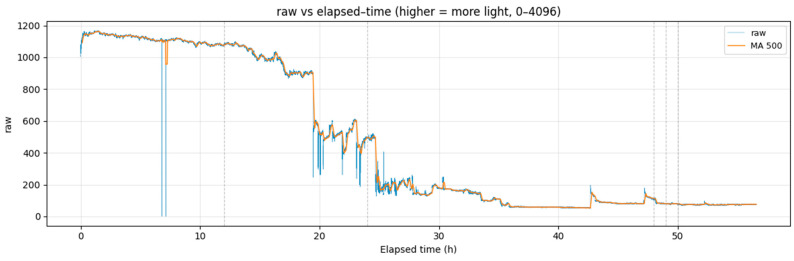
Raw photoresistor ADC signal recorded during successive LED measurements.

The recorded ADC values were grouped into time series corresponding to each conductive path length and processed in Python 3.14.3. A moving average filter was applied to the raw signals to reduce high-frequency noise, while data were plotted using Matplotlib 3.10.0 (Matplotlib Development Team, open-source software) to visualize the evolution of light intensity over time. Figure 17 presents an example of raw data acquired during the measurements.

The recorded signal showed clear differences between the investigated conductive path lengths. The step changes observed in Figure 17 correspond to the successive measurements performed on paths of different lengths, while the slower variations within each measurement interval indicate changes in light intensity during operation. Longer conductive paths were associated with lower LED brightness, consistent with the higher voltage drop expected from increased path resistance.

Overall, the case study showed that the electrical resistance of 3D-printed conductive paths can produce visible differences in LED brightness. The observed variations in the photoresistor signal are consistent with the practical relevance of the apparent resistivity changes measured on the straight specimens. However, the case study should be interpreted as a qualitative application-level demonstration, not as a separate quantitative validation of drift.

**Figure 18:**
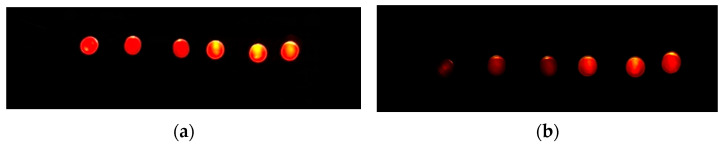
Qualitative visual illustration of LED brightness at t0 (**a**) and t50 (**b**).

The photographs in Figure 18 are included only as qualitative visual illustrations of the LED brightness at two measurement moments, t0 and t50. The experimental setup was not calibrated for absolute photometric measurements; therefore, luminous flux values in lumens were not reported. Since the visual appearance can be affected by camera focus, exposure, and positioning, the case study interpretation was based on the recorded photoresistor ADC signal shown in Figure 17, rather than on direct image comparison.

## 5. Conclusions and Further Work

The study investigated the influence of printing temperature, printing speed, and layer height on the apparent resistivity, repeatability, and time-dependent drift of a commercially available conductive PLA filament (ProtoPasta) over a 0–50 h interval. The main results can be summarized as follows:

- Printing temperature was the main factor controlling the initial apparent resistivity, with higher temperatures leading to lower resistivity.
- Layer height also affected the electrical response and it was identified as the main parameter influencing the time-dependent drift.
- Printing speed did not produce any significant change in the mean apparent resistivity within the tested range, although it influenced the specimen-to-specimen variability.
- Repeatability analysis showed that the robustness of the electrical response depends on both process parameters and time after fabrication. Several parameter combinations showed low variability immediately after printing, but the dispersion between replicates increased after 24 h and became more dependent on layer height.
- The analyzed process parameters affected different aspects of the electrical performance of MEX prints made of ProtoPasta: printing temperature mainly affected the initial resistivity level, while layer height and the layer height × temperature interaction were mainly associated with the time-dependent stability of apparent resistivity.

The case study on LEDs powered through 3D-printed conductive paths of different lengths supported the practical relevance of these observations. The gradual decrease in LED brightness suggested that time-dependent electrical changes can also occur in applications with prints of simple geometry made from the same conductive filament. However, this case study should be interpreted only as a qualitative application-level demonstration, not as a calibrated photometric validation.

All these results indicate that parameter selection based only on initial post-print measurements may not ensure stable electrical behavior in time. Thus, for MEX parts made of conductive filaments, not only the initial apparent resistivity, but also repeatability and post-print stability should be considered. This aspect is relevant for both research and practical applications, especially for low-current conductive paths, simple printed circuits, sensors, wearable devices, and embedded conductive features, where stable electrical behavior is required after 3D printing.

Future work will include studies on aging conditions, such as humidity, temperature cycling, and repeated electrical loading. A repeated-measures or mixed-effects statistical model could also be applied in future work to provide a more detailed description of the temporal evolution of apparent resistivity. Also, research is needed to compare two-probe and four-probe measurement configurations, evaluate other commercial conductive filaments, and develop functional sensor or circuit geometries with directly integrated conductive paths.

## Figures and Tables

**Table 2:** Investigated process parameters and fixed printing settings.

Parameter Levels	Fixed Process Parameters
Printing temperature: 210 °C, 220 °C, 230 °C Layer height: 0.2 mm, 0.3 mm, 0.4 mm Printing speed: 60 mm/s, 80 mm/s	Filament diameter: 1.75 mm Nozzle diameter: 0.4 mm Build plate adhesion: none Wall loops: 3 Raster orientation: 0° Infill pattern: line Infill density: 100% Bed temperature: 60 °C Fan speed: 100% Flow rate: 100% Line width: 0.4 mm Top/bottom layers: 1

**Table 3:** OrcaSlicer speed settings.

Global Settings		Per-Object Setting	
Initial layer	80 mm/s	Outer wall	60 mm/s
Outer wall	80 mm/s	Inner wall	60 mm/s
Inner wall	80 mm/s		

**Table 4:** Apparent resistivity measurements per specimen at t0.

Layer Height [mm]	Temperature [°C]	Apparent Resistivity [kΩ·mm]					
		80-1	80-2	80-3	60-1	60-2	60-3
0.2	210	0.187	0.197	0.194	0.191	0.192	0.195
	220	0.175	0.174	0.174	0.181	0.183	0.180
	230	0.163	0.169	0.173	0.169	0.180	0.175
0.3	210	0.174	0.194	0.205	0.173	0.190	0.188
	220	0.163	0.164	0.171	0.163	0.165	0.173
	230	0.157	0.170	0.165	0.172	0.170	0.170
0.4	210	0.178	0.187	0.192	0.180	0.188	0.193
	220	0.167	0.199	0.190	0.177	0.184	0.180
	230	0.156	0.173	0.176	0.159	0.174	0.174

**Table 5:** Specimen-to-specimen repeatability at t0.

Temperature [°C]	Layer Height [mm]	Apparent Resistivity [kΩ·mm] at 60 mm/s Printing Speed—Mean ± SD	Apparent Resistivity [kΩ·mm] at 80 mm/s Printing Speed—Mean ± SD
210	0.2	0.1927 ± 0.0021	0.1927 ± 0.0051
220	0.2	0.1813 ± 0.0015	0.1743 ± 0.0006
230	0.2	0.1747 ± 0.0055	0.1683 ± 0.0050
210	0.3	0.1837 ± 0.0093	0.1910 ± 0.0157
220	0.3	0.1670 ± 0.0053	0.1660 ± 0.0044
230	0.3	0.1707 ± 0.0012	0.1640 ± 0.0066
210	0.4	0.1870 ± 0.0066	0.1857 ± 0.0071
220	0.4	0.1803 ± 0.0035	0.1853 ± 0.0165
230	0.4	0.1690 ± 0.0087	0.1683 ± 0.0108

**Table 6:** ANOVA for apparent resistivity at t0.

Source	DF	SS	MS	F	P
Layer Height [mm]	2	0.000486	0.000243	3.87	0.028
Temperature [°C]	2	0.003588	0.001794	28.53	0.000
Speed [mm/s]	1	0.000019	0.000019	0.30	0.585
Error	48	0.003018	0.000063		
Total	53	0.007111			

**Table 7:** ANOVA for apparent resistivity at t50.

Source	DF	SS	MS	F	P
Layer Height [mm]	2	0.001047	0.000524	2.73	0.075
Temperature [°C]	2	0.001589	0.000794	4.15	0.022
Speed [mm/s]	1	0.000000	0.000000	0.00	0.961
Error	48	0.009195	0.000192		
Total	53	0.011831			

**Table 8:** Summary of the factorial ANOVA model for apparent resistivity drift (Δ*ρ*%) from t0 to t50.

Source	DF	*p*-Value
Layer height	2	0.000274
Temperature	2	0.0738
Speed	1	0.704
Layer height × Temperature	4	0.000038
Layer height × Speed	2	0.813
Temperature × Speed	2	0.601

## Data Availability

The original contributions presented in this study are included in the article/Supplementary Material. Further inquiries can be directed to the corresponding author.

## Supplementary Materials

The following supporting information can be downloaded at: https://www.mdpi.com/article/10.3390/polym18111274/s1, Table S1: Raw apparent resistivity results at t0; Table S2: Raw apparent resistivity results at t24; Table S3: Raw apparent resistivity results at t48; Table S4: Raw apparent resistivity results at t49; Table S5: Raw apparent resistivity results at t50; Table S6: Mean apparent resistivity and CV at t0 for specimens printed at 80 mm/s; Table S7: Mean apparent resistivity and CV at t0 for specimens printed at 60 mm/s; Table S8: Mean apparent resistivity and CV at t24 for specimens printed at 80 mm/s; Table S9: Mean apparent resistivity and CV at t24 for specimens printed at 60 mm/s; Table S10: Mean apparent resistivity and CV at t48 for specimens printed at 80 mm/s; Table S11: Mean apparent resistivity and CV at t48 for specimens printed at 60 mm/s; Table S12: Mean apparent resistivity and CV at t49 for specimens printed at 80 mm/s; Table S13: Mean apparent resistivity and CV at t49 for specimens printed at 60 mm/s; Table S14: Mean apparent resistivity and CV at t50 for specimens printed at 80 mm/s; Table S15: Mean apparent resistivity and CV at t50 for specimens printed at 60 mm/s.
