# Supplementary material for: Influence of 3D Printing Parameters on Apparent Resistivity, Repeatability and Time-Dependent Drift of Conductive PLA
Source: Polymers (Basel). 2026 May 22;18(11):1274. doi: 10.3390/polym18111274 (PMC13259191; doi:10.3390/polym18111274)
Supplement: Supplementary file 1 [file polymers-18-01274-s001.zip › polymers-4316361-supplementary.pdf]

Influence of 3D Printing Parameters on Apparent Resistivity, Repeatability and Time-Dependent Drift of Conductive PLA

SUPPLEMENTARY MATERIAL

Diana Popescu *, Ștefan Cula and Lidia Florentina Parpală

Department of Robots and Production Systems, National University of Science and Technology POLITEHNICA Bucharest, 060042 Bucharest, Romania; stefan.cula@upb.ro (Ș.C.); lidia.parpala@upb.ro (L.F.P.)
* Correspondence: diana.popescu@upb.ro

Table S1: Raw apparent resistivity results at t0

t0							
Layer Height [mm]	Temperature [degree C]	Apparent resistivity [kΩ /100mm]					
		80-1	80-2	80-3	60-1	60-2	60-3
0.2 [mm]	210	0.187	0.197	0.194	0.191	0.192	0.195
	220	0.175	0.174	0.174	0.181	0.183	0.180
	230	0.163	0.169	0.173	0.169	0.180	0.175
0.3 [mm]	210	0.174	0.194	0.205	0.173	0.190	0.188
	220	0.163	0.164	0.171	0.163	0.165	0.173
	230	0.157	0.170	0.165	0.172	0.170	0.170
0.4 [mm]	210	0.178	0.187	0.192	0.180	0.188	0.193
	220	0.167	0.199	0.190	0.177	0.184	0.180
	230	0.156	0.173	0.176	0.159	0.174	0.174

Table S2: Raw apparent resistivity results at t24

t24							
Layer Height [mm]	Temperature [degree C]	Apparent resistivity [kΩ /100mm]					
		80-1	80-2	80-3	60-1	60-2	60-3
0.2 [mm]	210	0.190	0.206	0.201	0.195	0.196	0.197
	220	0.181	0.180	0.177	0.181	0.189	0.183
	230	0.171	0.176	0.187	0.190	0.183	0.183
0.3 [mm]	210	0.181	0.201	0.217	0.168	0.190	0.181
	220	0.174	0.192	0.183	0.181	0.183	0.190
	230	0.176	0.181	0.178	0.179	0.166	0.206
0.4 [mm]	210	0.178	0.184	0.187	0.176	0.187	0.190
	220	0.166	0.158	0.187	0.171	0.177	0.179
	230	0.154	0.179	0.181	0.162	0.194	0.183

Table S3: Raw apparent resistivity results at t48

t48								
Layer Height [mm]	Temperature [degree C]	Apparent resistivity [kΩ /100mm]						
		80-1	80-2	80-3	60-1	60-2	60-3
0.2 [mm]	210	0.196	0.216	0.208	0.200	0.202	0.201
	220	0.193	0.188	0.181	0.195	0.200	0.189
	230	0.170	0.166	0.193	0.173	0.179	0.179
0.3 [mm]	210	0.185	0.202	0.220	0.172	0.194	0.183
	220	0.171	0.212	0.188	0.186	0.193	0.212
	230	0.186	0.197	0.193	0.187	0.174	0.221
0.4 [mm]	210	0.178	0.184	0.188	0.179	0.188	0.200
	220	0.161	0.178	0.181	0.166	0.173	0.171
	230	0.166	0.187	0.187	0.166	0.193	0.188

Table S4: Raw apparent resistivity results at t49

t49							
Layer Height [mm]	Temperature [degree C]	Apparent resistivity [kΩ /100mm]					
		80-1	80-2	80-3	60-1	60-2	60-3
0.2 [mm]	210	0.195	0.217	0.208	0.200	0.201	0.200
	220	0.193	0.189	0.181	0.193	0.195	0.190
	230	0.163	0.160	0.189	0.174	0.179	0.175
0.3 [mm]	210	0.187	0.197	0.221	0.170	0.192	0.181
	220	0.168	0.211	0.189	0.181	0.193	0.210
	230	0.181	0.183	0.175	0.174	0.167	0.218
0.4 [mm]	210	0.183	0.189	0.190	0.182	0.191	0.202
	220	0.162	0.179	0.182	0.166	0.173	0.172
	230	0.167	0.190	0.189	0.168	0.195	0.188

Table S5: Raw apparent resistivity results at t50

t50							
Layer Height [mm]	Temperature [degree C]	Apparent resistivity [kΩ /100mm]					
		80-1	80-2	80-3	60-1	60-2	60-3
0.2 [mm]	210	0.195	0.217	0.209	0.200	0.202	0.200
	220	0.194	0.191	0.185	0.196	0.196	0.191
	230	0.164	0.162	0.198	0.179	0.183	0.177
0.3 [mm]	210	0.190	0.199	0.219	0.171	0.194	0.182
	220	0.172	0.217	0.193	0.191	0.202	0.222
	230	0.184	0.186	0.178	0.176	0.169	0.221
0.4 [mm]	210	0.184	0.188	0.191	0.183	0.192	0.203
	220	0.164	0.180	0.183	0.167	0.175	0.173
	230	0.169	0.191	0.190	0.168	0.196	0.189

Table S6: Mean apparent resistivity and CV at t0 for specimens printed at 80 mm/s

t0, 80mm/s

Configuration (T / Speed / Layer)	Mean [kΩ/100 mm]	CV [%]± [kΩ/100 mm]	Notes
**210°C / 80 / 0.2 mm**	0.1927	2.66 ± 0.0050	Good repeatability
**220°C / 80 / 0.2 mm**	0.1743	0.33 ± 0.0005	Practically identical specimens
**230°C / 80 / 0.2 mm**	0.1683	2.99 ± 0.0050	Good repeatability
**210°C / 80 / 0.3 mm**	0.1910	8.23 ± 0.0155	One replicate clearly lower
**220°C / 80 / 0.3 mm**	0.1660	2.63 ± 0.0040	Good repeatability
**230°C / 80 / 0.3 mm**	0.1640	4.00 ± 0.0065	Moderate variability
**210°C / 80 / 0.4 mm**	0.1857	3.82 ± 0.0070	Moderate variability
**220°C / 80 / 0.4 mm**	0.1853	8.90 ± 0.0160	One replicate clearly lower
**230°C / 80 / 0.4 mm**	0.1683	6.41 ± 0.0100	One replicate clearly lower

Table S7: Mean apparent resistivityand CV at t0 for specimens printed at 60 mm/s

t0, 60mm/s

Configuration (T / Speed / Layer)	Mean [kΩ/100 mm]	CV [%] ± half-range [kΩ/100 mm]	Observations
**210°C / 60 / 0.2 mm**	0.1927	1.08 ± 0.0020	Good repeatability
**220°C / 60 / 0.2 mm**	0.1813	0.84 ± 0.0015	Practically identical specimens
**230°C / 60 / 0.2 mm**	0.1747	3.15 ± 0.0055	Moderate variability
**210°C / 60 / 0.3 mm**	0.1837	5.06 ± 0.0085	Moderate variability
**220°C / 60 / 0.3 mm**	0.1670	3.17 ± 0.0050	Moderate variability
**230°C / 60 / 0.3 mm**	0.1707	0.68 ± 0.0010	Practically identical specimens
**210°C / 60 / 0.4 mm**	0.1870	3.51 ± 0.0065	Moderate variability
**220°C / 60 / 0.4 mm**	0.1803	1.95 ± 0.0035	Good repeatability
**230°C / 60 / 0.4 mm**	0.1690	5.12 ± 0.0075	Moderate variability

Table S8: Mean apparent resistivityand CV at t24 for specimens printed at 80 mm/s

t24, 80mm/s

Configuration (T / Speed / Layer)	Mean [kΩ/100 mm]	CV [%] ± half-range [kΩ/100 mm]	Notes
**210°C / 80 / 0.2 mm**	0.1990	4.11 ± 0.0080	Moderate variability
**220°C / 80 / 0.2 mm**	0.1793	1.16 ± 0.0020	Good repeatability
**230°C / 80 / 0.2 mm**	0.1780	4.60 ± 0.0080	Moderate variability
**210°C / 80 / 0.3 mm**	0.1997	9.03 ± 0.0180	One replicate clearly lower
**220°C / 80 / 0.3 mm**	0.1830	4.92 ± 0.0090	Moderate variability
**230°C / 80 / 0.3 mm**	0.1783	1.41 ± 0.0025	Good repeatability
**210°C / 80 / 0.4 mm**	0.1830	2.50 ± 0.0045	Good repeatability
**220°C / 80 / 0.4 mm**	0.1703	8.79 ± 0.0145	One replicate clearly higher
**230°C / 80 / 0.4 mm**	0.1713	8.78 ± 0.0135	One replicate clearly lower

Table S9: Mean apparent resistivityand CV at t24 for specimens printed at 60 mm/s

t24, 60mm/s

Configuration (T / Speed / Layer)	Mean [kΩ/100 mm]	CV [%] ± half-range [kΩ/100 mm]	Notes
**210°C / 60 / 0.2 mm**	0.1960	0.51 ± 0.0010	Practically identical specimens
**220°C / 60 / 0.2 mm**	0.1843	2.26 ± 0.0040	Good repeatability
**230°C / 60 / 0.2 mm**	0.1853	2.18 ± 0.0035	Good repeatability
**210°C / 60 / 0.3 mm**	0.1797	6.16 ± 0.0110	One replicate clearly lower
**220°C / 60 / 0.3 mm**	0.1847	2.56 ± 0.0045	Good repeatability
**230°C / 60 / 0.3 mm**	0.1837	11.11 ± 0.0200	One replicate clearly higher
**210°C / 60 / 0.4 mm**	0.1843	4.00 ± 0.0070	Good repeatability
**220°C / 60 / 0.4 mm**	0.1757	2.37 ± 0.0040	Good repeatability
**230°C / 60 / 0.4 mm**	0.1797	9.05 ± 0.0160	One replicate clearly lower

Table S10: Mean apparent resistivityand CV at t48 for specimens printed at 80 mm/s

t48, 80mm/s

Configuration (T / Speed / Layer)	Mean [kΩ/100 mm]	CV [%] ± half-range [kΩ/100 mm]	Notes
**210°C / 80 / 0.2 mm**	0.2067	4.87 ± 0.0100	Moderate variability
**220°C / 80 / 0.2 mm**	0.1873	3.22 ± 0.0060	Moderate variability
**230°C / 80 / 0.2 mm**	0.1763	8.26 ± 0.0135	One replicate clearly higher
**210°C / 80 / 0.3 mm**	0.2023	8.65 ± 0.0175	High variability
**220°C / 80 / 0.3 mm**	0.1903	10.82 ± 0.0205	High variability
**230°C / 80 / 0.3 mm**	0.1920	2.90 ± 0.0055	Good repeatability
**210°C / 80 / 0.4 mm**	0.1833	2.75 ± 0.0050	Good repeatability
**220°C / 80 / 0.4 mm**	0.1733	6.22 ± 0.0100	One replicate clearly lower
**230°C / 80 / 0.4 mm**	0.1800	6.74 ± 0.0105	One replicate clearly lower

Table S11: Mean apparent resistivityand CV at t48 for specimens printed at 60 mm/s

t48, 60mm/s

Configuration (T / Speed / Layer)	Mean [kΩ/100 mm]	CV [%] ± half-range [kΩ/100 mm]	Notes
**210°C / 60 / 0.2 mm**	0.2010	0.50 ± 0.0010	Practically identical specimens
**220°C / 60 / 0.2 mm**	0.1947	2.83 ± 0.0055	Good repeatability
**230°C / 60 / 0.2 mm**	0.1770	1.96 ± 0.0030	Good repeatability
**210°C / 60 / 0.3 mm**	0.1830	6.01 ± 0.0110	High variability
**220°C / 60 / 0.3 mm**	0.1970	6.83 ± 0.0130	High variability
**230°C / 60 / 0.3 mm**	0.1940	12.51 ± 0.0235	High variability (spread across all three)
**210°C / 60 / 0.4 mm**	0.1890	5.57 ± 0.0105	Moderate variability
**220°C / 60 / 0.4 mm**	0.1700	2.12 ± 0.0035	Good repeatability
**230°C / 60 / 0.4 mm**	0.1823	7.88 ± 0.0135	One replicate clearly lower

Table S12: Mean apparent resistivityand CV at t49 for specimens printed at 80 mm/s

t49, 80mm/s

Configuration (T / Speed / Layer)	Mean [kΩ/100 mm]	CV [%] ± half-range [kΩ/100 mm]	Notes
**210°C / 80 / 0.2 mm**	0.2067	5.35 ± 0.0110	Moderate variability
**220°C / 80 / 0.2 mm**	0.1877	3.26 ± 0.0060	Moderate variability
**230°C / 80 / 0.2 mm**	0.1707	9.34 ± 0.0145	One replicate clearly higher
**210°C / 80 / 0.3 mm**	0.2017	8.66 ± 0.0170	High variability
**220°C / 80 / 0.3 mm**	0.1893	11.36 ± 0.0215	One replicate clearly lower
**230°C / 80 / 0.3 mm**	0.1797	2.32 ± 0.0040	Good repeatability
**210°C / 80 / 0.4 mm**	0.1873	2.02 ± 0.0035	Good repeatability
**220°C / 80 / 0.4 mm**	0.1743	6.19 ± 0.0100	One replicate clearly lower
**230°C / 80 / 0.4 mm**	0.1820	7.14 ± 0.0115	One replicate clearly lower

Table S13: Mean apparent resistivityand CV at t49 for specimens printed at 60 mm/s

t49, 60mm/s

Configuration (T / Speed / Layer)	Mean [kΩ/100 mm]	CV [%] ± half-range [kΩ/100 mm]	Notes
**210°C / 60 / 0.2 mm**	0.2003	0.29 ± 0.0005	Practically identical specimens
**220°C / 60 / 0.2 mm**	0.1927	1.31 ± 0.0025	Good repeatability
**230°C / 60 / 0.2 mm**	0.1760	1.50 ± 0.0025	Good repeatability
**210°C / 60 / 0.3 mm**	0.1810	6.08 ± 0.0110	One replicate clearly lower
**220°C / 60 / 0.3 mm**	0.1947	7.49 ± 0.0145	One replicate clearly higher
**230°C / 60 / 0.3 mm**	0.1863	14.84 ± 0.0255	One replicate clearly higher
**210°C / 60 / 0.4 mm**	0.1917	5.23 ± 0.0100	Moderate variability
**220°C / 60 / 0.4 mm**	0.1703	2.22 ± 0.0035	Good repeatability
**230°C / 60 / 0.4 mm**	0.1837	7.63 ± 0.0135	One replicate clearly lower

Table S14: Mean apparent resistivityand CV at t50 for specimens printed at 80 mm/s

t50, 80mm/s

Configuration (T / Speed / Layer)	Mean [kΩ/100 mm]	CV [%] ± half-range [kΩ/100 mm]	Notes
**210°C / 80 / 0.2 mm**	0.2070	5.38 ± 0.0110	Moderate variability
**220°C / 80 / 0.2 mm**	0.1900	2.41 ± 0.0045	Good repeatability
**230°C / 80 / 0.2 mm**	0.1747	11.58 ± 0.0180	One replicate clearly higher
**210°C / 80 / 0.3 mm**	0.2027	7.32 ± 0.0145	High variability
**220°C / 80 / 0.3 mm**	0.1940	11.61 ± 0.0225	One replicate clearly lower
**230°C / 80 / 0.3 mm**	0.1827	2.28 ± 0.0040	Good repeatability
**210°C / 80 / 0.4 mm**	0.1877	1.87 ± 0.0035	Good repeatability
**220°C / 80 / 0.4 mm**	0.1757	5.81 ± 0.0095	Moderate variability
**230°C / 80 / 0.4 mm**	0.1833	6.78 ± 0.0110	One replicate clearly lower

Table S15: Mean apparent resistivityand CV at t50 for specimens printed at 60 mm/s

t50, 60mm/s

Configuration (T / Speed / Layer)	Mean [kΩ/100 mm]	CV [%] ± half-range [kΩ/100 mm]	Notes
**210°C / 60 / 0.2 mm**	0.2007	0.58 ± 0.0010	Practically identical specimens
**220°C / 60 / 0.2 mm**	0.1943	1.49 ± 0.0025	Good repeatability
**230°C / 60 / 0.2 mm**	0.1797	1.70 ± 0.0030	Good repeatability
**210°C / 60 / 0.3 mm**	0.1823	6.31 ± 0.0115	One replicate clearly lower
**220°C / 60 / 0.3 mm**	0.2050	7.67 ± 0.0155	One replicate clearly higher
**230°C / 60 / 0.3 mm**	0.1887	14.96 ± 0.0260	One replicate clearly higher
**210°C / 60 / 0.4 mm**	0.1927	5.20 ± 0.0100	Moderate variability
**220°C / 60 / 0.4 mm**	0.1717	2.43 ± 0.0040	Good repeatability
**230°C / 60 / 0.4 mm**	0.1843	7.91 ± 0.0140	One replicate clearly lower
